# Preprocessing surface EMG data removes voluntary muscle activity and enhances SPiQE fasciculation analysis

**DOI:** 10.1016/j.clinph.2019.09.015

**Published:** 2020-01

**Authors:** J. Bashford, A. Wickham, R. Iniesta, E. Drakakis, M. Boutelle, K. Mills, CE. Shaw

**Affiliations:** a UK Dementia Research Institute, Department of Basic and Clinical Neuroscience, Maurice Wohl Clinical Neuroscience Institute, Institute of Psychiatry, Psychology and Neuroscience, King’s College London, UK; b Department of Bioengineering, Imperial College London, UK; c Department of Biostatistics and Health Informatics, King’s College London, UK

**Keywords:** Amyotrophic lateral sclerosis, Fasciculation, High-density surface EMG, Biomarker, Motor unit, ALS, amyotrophic lateral sclerosis, AVID, active voluntary identification, BFS, benign fasciculation syndrome, (HD)SEMG, (high-density) surface electromyography, IQR, inter-quartile range, NPV, negative predictive value, PPV, positive predictive value, SD, standard deviation, SPiQE, Surface Potential Quantification Engine

## Abstract

**Objectives:**

Fasciculations are a clinical hallmark of amyotrophic lateral sclerosis (ALS). The Surface Potential Quantification Engine (SPiQE) is a novel analytical tool to identify fasciculation potentials from high-density surface electromyography (HDSEMG). This method was accurate on relaxed recordings amidst fluctuating noise levels. To avoid time-consuming manual exclusion of voluntary muscle activity, we developed a method capable of rapidly excluding voluntary potentials and integrating with the established SPiQE pipeline.

**Methods:**

Six ALS patients, one patient with benign fasciculation syndrome and one patient with multifocal motor neuropathy underwent monthly thirty-minute HDSEMG from biceps and gastrocnemius. In MATLAB, we developed and compared the performance of four Active Voluntary IDentification (AVID) strategies, producing a decision aid for optimal selection.

**Results:**

Assessment of 601 one-minute recordings permitted the development of sensitive, specific and screening strategies to exclude voluntary potentials. Exclusion times (0.2–13.1 minutes), processing times (10.7–49.5 seconds) and fasciculation frequencies (27.4–71.1 per minute) for 165 thirty-minute recordings were compared. The overall median fasciculation frequency was 40.5 per minute (10.6–79.4 IQR).

**Conclusion:**

We hereby introduce AVID as a flexible, targeted approach to exclude voluntary muscle activity from HDSEMG recordings.

**Significance:**

Longitudinal quantification of fasciculations in ALS could provide unique insight into motor neuron health.

## Highlights

• A novel preprocessing step removes the need for manual selection of relaxed surface EMG data.
• SPiQE provides reliable fasciculation analysis from raw thirty-minute recordings in ALS.
• This paves the way for clinical calibration of a potential novel biomarker of disease progression.

## 1 Introduction

ALS causes progressive neurological weakness due to motor neuron degeneration, typically leading to death within four years of symptom onset (Al-Chalabi and Hardiman, 2013). It has a lifetime risk of 1 in approximately 400 and there is an urgent need for new disease-modifying therapies (Alonso et al., 2009, Petrov et al., 2017). Accessible, validated biomarkers of disease activity offer an opportunity to inform and guide the selection, stratification and outcomes of patients in clinical trials (Benatar et al., 2016).

A fasciculation can be defined as an involuntary muscle twitch arising from the random, spontaneous activation of a single motor unit (de Carvalho et al., 2017). Fasciculations are a hallmark clinical feature of ALS, reflecting the pathological hyperexcitability of motor neurons in this disorder (Gordon, 2013, De Carvalho and Swash, 2016b). Due to their presence from the earliest stages of disease, the systematic quantification of fasciculation potentials over time may enhance our understanding of motor neuron health in ALS (de Carvalho et al., 2017). The non-invasive nature of high-density surface EMG (HDSEMG) increases patient tolerance to investigation and generates massive data sets in an objective and systematic way (Zwarts and Stegeman, 2003). We recently developed the Surface Potential Quantification Engine (SPiQE) as a novel analytical tool capable of automatically and accurately identifying fasciculation potentials from extended HDSEMG recordings that can be repeated in longitudinal studies (Bashford et al., 2019).

We recognised two principle sources of error in this setting. The first was noise (De Luca et al., 2010), which has been discussed previously (Bashford et al., 2019). The second was the presence of voluntary muscle activity, represented by the regular firing of motor unit potentials on EMG. This is problematic in other recordings of resting electrographic signals, such as electroencephalography (Li et al., 2018). If not appropriately addressed, significant errors in fasciculation analysis could appear due to the higher firing rate of voluntary potentials compared to fasciculation potentials.

Automated analytical methods aim to reduce the dependency on human decision-making, thereby limiting error, bias and time input (Kini et al., 2016, Roldan-Vasco et al., 2018). We sought to develop a method that was as automated and user-friendly as possible to account for the full range of data quality and content. For this approach to work in practice, the system would need to provide easily interpretable readouts to guide each stage of the analysis.

Machine learning can be used to classify labels based on extracted features from any data set (Sakai and Yamada, 2019, Rush et al., 2019, Roldan-Vasco et al., 2018, Zhang et al., 2014). The model can be trained with a proportion of data (e.g. 80%) and tested on the remainder. Classification accuracy within the test set is a less biased estimate of the model’s performance (Kim, 2009). Bootstrap aggregation (‘bagging’) is designed to make the predictive model less volatile through an averaging process (Kohavi, 1995). We set out to employ these methods in the design and implementation of the analytical tool that we have named Active Voluntary IDentification (AVID). Specifically, we hypothesised that *falsely identified* voluntary potentials could be automatically distinguished from *true* voluntary potentials, allowing for their correct inclusion in the main fasciculation analysis.

By integrating AVID into the established SPiQE pipeline, we were able to analyse the full thirty-minute recordings without prior manual processing. In the final step of this paper, we present the overall fasciculation frequencies of all recordings as an introduction to SPiQE’s practical utility as a quantitative tool. We anticipate that this will serve as the foundation to calibrate a range of clinical biomarkers with potential diagnostic, prognostic and disease-monitoring value.

## 2 Methods

### 2.1 Patient characteristics

Six patients with ALS, one patient with benign fasciculation syndrome and one patient with multifocal motor neuropathy underwent 42 assessments in total at intervals of at least one month (Table 1). ALS patients were diagnosed with probable (including laboratory-supported) or definite ALS using the revised El Escorial Criteria. Ethical approval was obtained from the North of Scotland Research Ethics Service (Ref: 15/NS/0103). Patients were recruited from the King’s College Hospital Motor Nerve Clinic between Jan-Feb 2016 and provided informed written consent before participation.

**Table 1:** **Patient characteristics.** ALS, amyotrophic lateral sclerosis; MMN, multifocal motor neuropathy; BFS, benign fasciculation syndrome. Muscle powers were assessed at baseline by the same clinician (JB). * Treated with intravenous immunoglobulin.

**Patient No.**	**Age (years)**	**Gender**	**Diagnosis**	**Site of symptom onset**	**Duration since symptom onset (months)**	**Biceps power (MRC scale, 5 = normal)**		**Gastrocnemius power (MRC scale, 5 = normal)**		**Number of assessments undertaken**
						**R**	**L**	**R**	**L**	
1	59	M	ALS	Left leg	24	5	5	5	5	4
2	57	M	ALS	Right arm	19	4	4	4	4	5
3	50	M	ALS	Left arm	23	2	2	5	5	4
4	58	M	ALS	Right arm	60	1	1	5	5	6
5	59	F	ALS	Bulbar	27	5	5	5	5	6
6	61	M	ALS	Left arm	10	5	5	5	5	7
7	68	F	MMN*	Left arm	204	4	5	5	5	6
8	57	M	BFS	Both legs	8	5	5	5	5	4

### 2.2 High-density surface EMG (HDSEMG)

At each assessment, 30-minute HDSEMG recordings were taken from biceps brachii and medial gastrocnemii muscles bilaterally. The sensor had 64 circular electrodes (Ag/AgCl; 8x8 grid; electrode diameter 4.5 mm; inter-electrode distance 8.5 mm; TMS International BV, The Netherlands). For biceps, the lower edge of the sensor was placed along a line approximately 2 cm above the antecubital fossa. For gastrocnemius, the center of the sensor was placed approximately a third of the way along a line from the popliteal fossa to the medial malleolus. The tail of the sensor was always positioned inferiorly. Linear measurements between the medial inferior corner of the grid and the medial epicondyle (for biceps) or malleolus (for gastrocnemius) guided sensor placement on subsequent visits for each patient. Prior to sensor application, the skin was lightly scrubbed with an abrasive gel and a 70% alcohol wipe. A template facilitated the application of conducting gel. Transpore surgical plastic tape (3 M UK PLC) was used to secure any edges of the sensor that did not adhere to the skin surface well. Reference electrodes (solid gel with snap connector; 35x45mm) were placed over the ipsilateral olecranon (for biceps) and dorsum of the foot (for gastrocnemius). Patients relaxed on the examination couch with legs in a horizontal, partially flexed position and forearms prone with an elbow angle of 90–120 degrees.

All signals from the 64 channels were amplified against the average of all connected unipolar inputs by the Refa-64 EMG Recording System (TMS International BV). A bandpass filter (20–500 Hz) was applied with 50 Hz notch filtering in MATLAB. The raw HDSEMG data (16-bit) were stored as proprietary Polybench (version 1.30; TMS International BV) files at a sampling rate of 2048 Hz per channel. Sensors were cleaned using propran-2-ol solution in the laboratory and re-used up to three times according to manufacturer guidance.

### 2.3 SPiQE pipeline

Description and validation of the SPiQE pipeline has been previously reported in detail (Bashford et al., 2019). In brief, we opted to universally discard the outer perimeter of channels to make the size of the dataset more manageable, having observed that the quality of signal amongst these channels was sometimes poor due to suboptimal skin-electrode contact at the edges (particularly for the more curved skin surface overlying gastrocnemius). An initial screen for motor unit potentials was applied to each of the central 36 channels (6x6 grid) that made up the HDSEMG recording. This involved the detection of the most extreme amplitudes (positive and negative) that occurred in less than 2% of the recording. These areas represented the peaks and troughs of motor unit potentials. For each of these potentials, the channel with the greatest peak-trough amplitude difference was transferred into a ‘super-channel’. This converted a 36-channel recording into a single-dimensional trace.

Based on manual counts, we previously found a linear relationship between average noise levels and the optimal amplitude threshold for fasciculation potential inclusion (Bashford et al., 2019). We confirmed that the optimal automated model was a noise-responsive algorithm, capable of adjusting its amplitude inclusion threshold (AT_inc_; Y) according to the local noise level (X; Y = 8X). In addition, areas of the recording with excessive noise were automatically identified and excluded from further analysis based on an optimal amplitude exclusion threshold (AT_exc_) of 100 μV. This was achieved by splitting recordings into blocks of five seconds duration. A block was excluded if either: (a) AT_inc_ exceeded the AT_exc_ for greater than half of identified potentials; or, (b) at least one AT_inc_ was greater than double the AT_exc_. When a block was excluded, five seconds were taken from the total time and the potentials detected within it were excluded. This pipeline achieved a classification accuracy of 88% when applied to 5,318 fasciculation potentials that had been identified manually.

### 2.4 Computation and statistical analysis

All computation of EMG data was performed in the analytical platform MATLAB (R2014a) using specifically designed scripts on laptops with Intel i7 (2.5 GHz) processors. Statistical tests were performed in Prism V7.0a. The Mann-Whitney test was used for non-parametric data.

### 2.5 Defining AVID using one-minute recording samples

#### 2.5.1 Manual categorisation of voluntary activity

Visits 1–4 were used for validation to ensure equal contributions from all eight patients. For each of these 32 assessments, five one-minute sample recordings starting at 5/10/15/20/25 minutes underwent manual inspection. The process for the identification of voluntary potentials was finalised prior to manual inspection to ensure consistency across the recordings:

a. The autoscale function in Polybench optimised the visualisation of motor unit potentials across the 64 channels for successive 10-second windows;
b. A motor unit potential was defined as a spike in ≥10 channels (/64) simultaneously;
c. The presence of multiple channels permitted the manual identification of motor unit potentials with identical morphologies;
d. A train of voluntary potentials was identified if at least four identical motor unit potentials were present with inter-spike intervals <250 ms. These thresholds were based on the lowest firing rate of motor unit recruitment (approximately 4 Hz) and the importance of not excluding multiplets (e.g. pairs) due to their potential pathological significance (Kleine et al., 2008, Sleutjes et al., 2015, De Luca et al., 1982, Broman et al., 1985).

This process permitted the categorisation of each one-minute recording into the following groups: 1. *Fully relaxed* (voluntary potentials were absent throughout the whole minute); 2. *Partially relaxed* (voluntary potentials were identified for less than one minute); 3. *Voluntarily active* (voluntary potentials were present throughout the whole minute); or, 4. *Excluded due to poor quality* (EMG contained excessive noise on visual inspection across all channels with no discernible spikes resembling typical motor unit activity, thereby preventing categorisation into one of the other three groups). For each of the ‘partially relaxed’ samples, the onset and offset of each voluntary spell was recorded. JB was the sole assessor.

#### 2.5.2 Automated detection of voluntary potentials

**Fig. 1:**
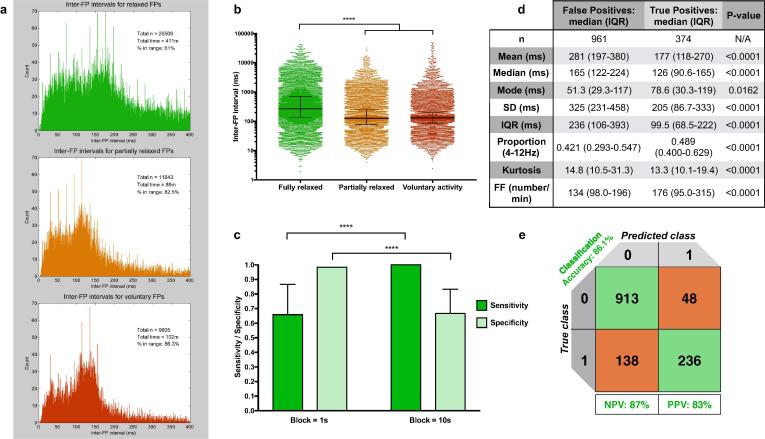
**Defining AVID using one-minute recording samples. a***Inter-fasciculation interval histograms between 0*–*400 ms for three groups of one-minute sample recordings:* fully relaxed (green), partially relaxed (orange) and voluntary activity (red). **b***Inter-fasciculation interval distributions for three categories.* Lines for median and IQR displayed. Note logarithmic scale on y-axis. **c***Sensitivity and specificity of chosen block duration* (10 s = AVID-1A; 1 s = AVID-2). Bar represents median value, whisker shows upper 95% confidence interval. **d***Comparison of eight extracted features from 10 s ‘voluntary’ blocks in AVID-1A,* highlighting significant differences between false and true positives. **e***Confusion matrix.* Predictive ability of the optimal classification model after five-fold cross-validation. ‘1′ represents ‘true’, ‘0′ represents ‘false’. FF, fasciculation frequency; IQR, inter-quartile range; NPV, negative predictive value; PPV, positive predictive value; SD, standard deviation. Mann-Whitney tests used.

The same one-minute recordings that had been used for manual categorisation were subsequently screened in an automated way for the presence of voluntary potentials. This involved splitting each one-minute recording into blocks of different durations (1–30 s). Any block that contained a train of four or more voluntary potentials was labeled as ‘voluntary’. Therefore, the longer the block duration, the more likely it would capture all the voluntary activity found manually. However, this would come at the expense of falsely identifying some sections as ‘voluntary’ that were found to be ‘relaxed’ on manual inspection. The extremely high sensitivity achieved with 10 s blocks represented a saturation point (Fig. 1c), and no further increase in sensitivity could be achieved by increasing the block size. Consequently, blocks of 1 s (high specificity and low sensitivity for voluntary potentials) and 10 s (low specificity and high sensitivity for voluntary potentials) were chosen as they represented two ends of the performance spectrum (see Section 3.1.2).

Due to the difficulty in defining a universal strategy, four separate AVID strategies were designed, named AVID-0, AVID-1A, AVID-1B and AVID-2:

0. AVID was disabled.
1. Sensitive approach, involving blocks of 10 s duration. This was split into:
  A. Unadjusted.
  B. Adjusted. The blocks identified in 1A as ‘voluntary’ underwent a classification process (see Sections 2.5.3 and 3.1.3) to distinguish true and false positives.
2. Specific approach, involving blocks of 1 s duration.

To determine the sensitivity and specificity of AVID-1A and -2, manual and automated results were compared. A true positive was recorded if a block had been assessed as containing voluntary activity by both manual and automated methods. True negatives, false positives and false negatives were recorded accordingly, allowing computation of the sensitivity and specificity (Linden, 2006).

#### 2.5.3 Refinement using a classification model

A classification model was used to adjust the results from AVID-1A, which was a highly sensitive but not very specific approach (Sakai and Yamada, 2019). The aim was to distinguish true and false positives, thereby enabling false positives to be returned to the analysable pool.

Using the manual labels for the ‘voluntary’ blocks detected by AVID-1A, a classification model was trained using eight extracted features related to intervals between successive potentials. Six features were based on the full range of intervals: median, mean, mode, standard deviation, inter-quartile range and fasciculation frequency. Two features were calculated from intervals between 84–250 ms (representing 4–12 Hz, the typical frequency of voluntary potentials at low force levels): kurtosis, and proportion of total intervals within this range. A 5-fold cross-validation process estimated the model’s predictive ability (Kim, 2009). Within MATLAB’s Classification Learner App, a default set of classifiers (including decision tree, discriminant analysis, support vector machine, nearest neighbor and ensemble methods) was applied and the optimal classifier was the one that produced the highest classification accuracy.

### 2.6 Testing AVID using thirty-minute recordings

#### 2.6.1 Data quality and exclusion

Channels were excluded as previously reported (Bashford et al., 2019). In brief, poorly behaving channels were excluded in an automated way. This comprised channels that were null due to absent electrical contact or those that contained excessive noise, artefacts or baseline drift. This involved performing the fast Fourier transform, analysing the area under the curve and calculating the amplitude range across the channel. Channels that fell outside the 95% confidence interval for any of these parameters were excluded.

A potential’s noise band was defined as the difference between the mean positive amplitude and the mean negative amplitude for one second both before and after the detected potential. The median of all the individual noise bands was defined as the noise output for the whole recording. Median was a more reliable measure of average noise than mean due to the occasional presence of large positive outliers.

#### 2.6.2 Optimal AVID strategy

**Fig. 2:**
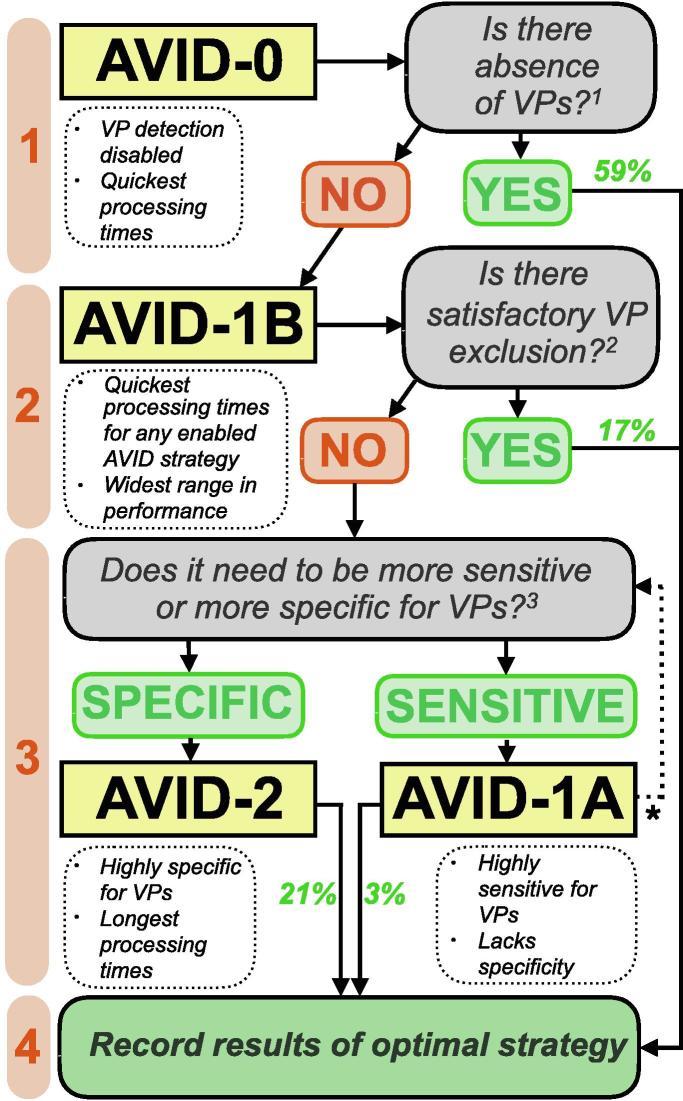
**Selection of optimal AVID strategy.***Decision aid.* Stages 2 and 3 were not required for 59% of recordings. Percentages highlight proportion of recordings that were optimal with the respective AVID strategy. Notable advantages and disadvantages of each strategy are highlighted. ^1^Manual interpretation of the inter-fasciculation interval histogram (see Fig. 3) is required. The answer is ‘Yes’ if there is no interval peak in the range of voluntary firing (100–200 ms); ^2^This requires manual interpretation of both the inter-fasciculation interval histogram (see Fig. 3) and the timeline of fasciculation and voluntary potential firing (see Fig. 4). The answer is ‘No’ if either: a) Voluntary potentials remain present; or, b) All voluntary potentials have been excluded at the expense of excluding large portions of ‘relaxed’ data. ^3^Answer ‘sensitive’ if option ‘a’ was selected at stage 2, answer ‘specific’ if option ‘b’ was selected. *When AVID-1A is not specific enough for voluntary potentials, it is necessary to return to start of stage 3 and compare with AVID-2.

**Fig. 3:**
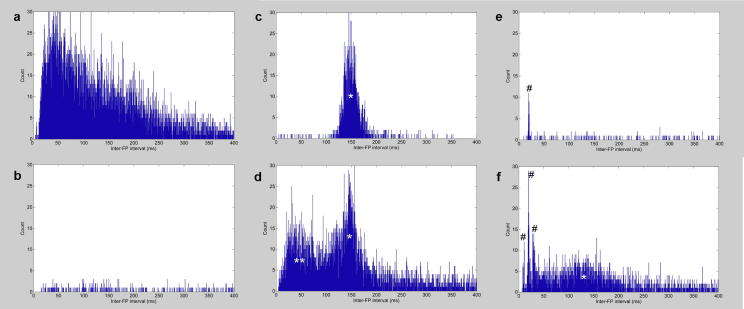
**Observed inter-fasciculation interval histograms.** Example outputs containing: **a** No voluntary potentials with high background fasciculation frequency. **b** No voluntary potentials with low background fasciculation frequency. **c** Voluntary potentials from a single active motor unit firing at 5–10 Hz. **d** Voluntary potentials from multiple active motor units. **e** Possible short interval multiplets (pairs/triplets). **f** A mixture of voluntary potentials and possible multiplets. * Interval peak consistent with regular firing of a single motor unit; ** Interval peak consistent with regular firing of multiple motor units simultaneously; # Interval peak consistent with possible multiplet activity (pairs/triplets) at varying intervals < 50 ms.

This was chosen after manual inspection of the inter-fasciculation interval histogram for the recording (see Section 3.2.2 for further details). A decision aid (Fig. 2) and representative inter-fasciculation interval histograms (Fig. 3) were used to interpret individual interval histograms, allowing for manual selection of the optimal AVID strategy for each recording. All selections were made by the same assessor (JB).

#### 2.6.3 Fasciculation frequency

The fasciculation frequency was calculated by dividing the total number of analysable fasciculation potentials by the total time that remained after any excluded epochs. Coefficients of determination (r^2^) between fasciculation frequency and data quality readouts (median noise band and number of excluded channels) were calculated.

## 3 Results

### 3.1 Defining AVID using one-minute recording samples

#### 3.1.1 Manual categorisation of voluntary activity

Out of 601 one-minute recording samples, 411 of them (68.3%) were fully relaxed containing 26,508 inter-fasciculation intervals, 88 (14.6%) were partially relaxed containing 11,843 intervals and 102 (17.0%) contained pure voluntary activity with 9,805 intervals (Fig. 1a). The median interval during relaxed recordings was greater than during recordings containing voluntary activity (267.1 vs. 129.9 ms, p < 0.0001, Mann-Whitney test; Fig. 1b). Voluntary activity caused a peak between 100–160 ms, consistent with regular motor unit firing at 6–10 Hz. A higher proportion of intervals < 400 ms were present during voluntary activity (86.3%) compared to relaxed recordings (61%).

#### 3.1.2 Automated detection of voluntary potentials

AVID-1A correctly identified 100% (median; 100–100% IQR) of the blocks that had been manually labeled as ‘voluntary’, while accurately identifying only 67% (median; 0–100% IQR) of the blocks that had been manually labeled as ‘relaxed’ (Fig. 1c)*.* In contrast, AVID-2 correctly identified 66% (median; 11–100% IQR) of the ‘voluntary’ blocks, while accurately identifying 98% (median; 88–100% IQR) of the ‘relaxed’ blocks. This meant AVID-1A was the most sensitive method for detecting voluntary activity, whereas AVID-2 was the most specific.

#### 3.1.3 Refinement using a classification model

A total of 1,335 blocks from AVID-1A were used to train and test the optimal predictive model (Fig. 1d-e). This was an ensemble bagged trees classifier with 30 learners and principle component analysis disabled. There were no clearly redundant features in the parallel coordinates plot within MATLAB’s Classification Learner App, therefore all eight features were kept in the model. Through 5-fold cross-validation, the estimated classification accuracy was 86.1%, the positive predictive value was 83.0% and the negative predictive value was 87.0%. Therefore, the classification model allowed falsely identified blocks to be automatically returned to the analysable pool with a reasonable degree of accuracy.

### 3.2 Testing AVID using thirty-minute recordings

#### 3.2.1 Data quality and exclusion

In total, 168 recordings were taken from eight patients at four muscular sites on 4–7 separate occasions. Three recordings were of too poor quality to be analysed. For the remaining 165 recordings, the average noise band was 3.18 μV (median; IQR 2.15–5.00). There were 16 (median; 13–19 IQR) out of 36 channels excluded per recording.

Across all 165 recordings, 0.2 minutes (median; 0.0–1.5 IQR) were excluded per recording due to excessively noisy periods (Table 2). This was independent of the AVID strategy used. For the 68 recordings with AVID enabled, an additional 4.5 minutes (median; 2.1–8.4 IQR) were excluded per recording. When considering the optimal AVID strategy (0/1A/1B/2) for all 165 recordings, 1.5 minutes (median; 0.0–6.5 IQR) were excluded per recording due to a combination of excessively noisy portions of data and/or the detection of voluntary muscle activity. Therefore, on average there remained 28.5 minutes per recording for fasciculation analysis.

**Table 2:** **Testing AVID using thirty-minute recordings.** Summary of results for each AVID strategy when applied to 165 recordings. Medians (inter-quartile range) are displayed. Square brackets indicate units or percentage. AT_exc_ 100, amplitude exclusion threshold 100 μV.

			**AVID strategy**			
			**0 (disabled)**	**1A**	**1B**	**2**
**Number of recordings**			165			
**SPiQE outputs**	**Time analysed [mins]**		29.8 (28.5–30.0)	16.9 (7.8–26.9)	27.7 (22.5–29.3)	26.6 (19.5–29.4)
	**Time excluded [mins]**	**AT_exc_ 100**	0.2 (0–1.5)			
		**AVID**	0.0 (0.0–0.0)	8.8 (1.8–18.9)	1.2 (0.1–3.9)	1.2 (0.2–6.0)
		**Total**	0.2 (0–1.5)	13.1 (3.1–22.2)	2.3 (0.7–7.5)	3.4 (0.6–10.5)
	**Detected FPs**	**Total number per recording**	2050 (447–3840)	273 (74–696)	958 (264–1740)	826 (260–1660)
		**Average frequency [num/min]**	71.1 (19.5–134)	27.4 (7.6–57.9)	38.8 (10.6–71.5)	38.8 (9.9–73.2)
**Processing time [s]**			10.7 (8.1–18.6)	36.1 (18.0–66.6)	16.3 (10.5–32.9)	49.5 (18.3–151.1)
**Number of recordings with this strategy as optimal [%]**			97 [58.8]	5 [3.0]	28 [17.0]	35 [21.2]

#### 3.2.2 Optimal AVID strategy

Out of 165 recordings, 97 of them (58.8%) were deemed optimal with AVID disabled (AVID-0), 33 (20.0%) with AVID 1A/1B and 35 (21.2%) with AVID 2 (Table 2).

**Fig. 4:**
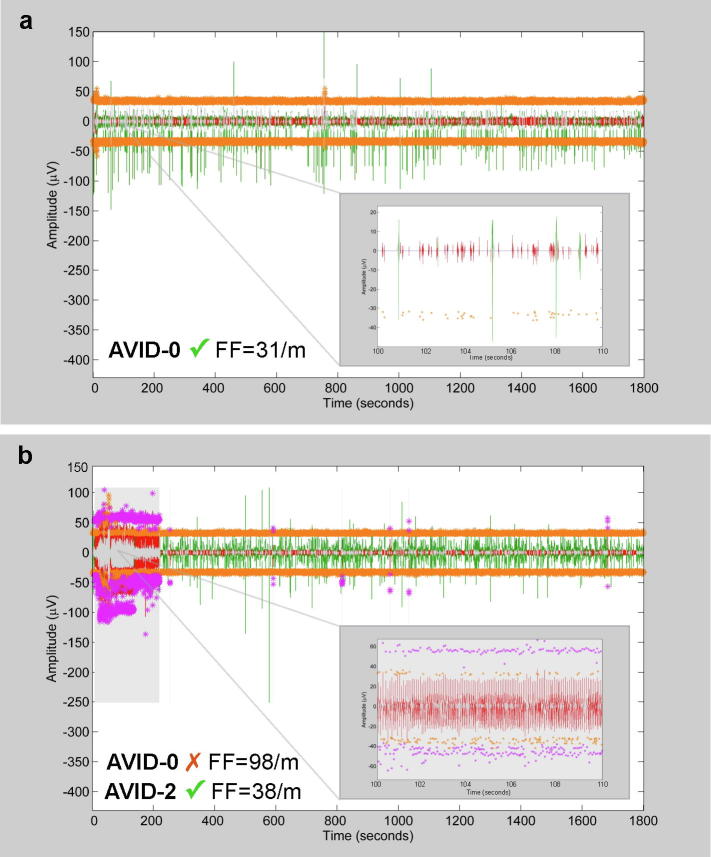
**Comparing AVID outputs. a***AVID-0 optimal.* Thirty-minute timeline of included fasciculation potentials (green) from the right biceps of an ALS patient. *Insert:* 10 s example demonstrating random temporal pattern of fasciculation potentials. Orange asterisks indicate excluded spikes that did not meet amplitude inclusion threshold. Note that closely aligned asterisks have merged into continuous orange lines when viewed over 1800s. **b***AVID-2 optimal.* Thirty-minute timeline of included fasciculation potentials (green) from the left gastrocnemius of an ALS patient. Shaded region indicates excluded epoch of voluntary activity. Effect of AVID strategy on fasciculation frequency displayed, underlying importance of choosing optimal strategy based on inter-fasciculation interval histograms (Fig. 2). *Insert:* 10 s example demonstrating regular temporal pattern of voluntary potentials. Purple asterisks indicate excluded potentials identified as voluntary. FF, fasciculation frequency. (For interpretation of the references to colour in this figure legend, the reader is referred to the web version of this article.)

Armed with detailed knowledge of the performance of each strategy, we devised a simplified decision aid for selecting the optimal strategy (Fig. 2). To progress between the different stages of the decision aid, manual interpretation of the inter-fasciculation interval histogram was required. Reference to template histograms (Fig. 3) enabled the user (JB) to decide whether voluntary activity was present at baseline (AVID-0). If absent, then the results from AVID-0 (fasciculation frequency, time included etc.) were recorded. Otherwise, each subsequent stage required further manual interpretation of the interval histogram (related to the relevant AVID strategy) to work out which strategy was optimal at excluding voluntary activity. This decision aid aimed to maximise the number of analysed fasciculation potentials, while optimising sensitivity/specificity and minimising the processing time. This approach should achieve high objectivity and enable direct comparisons between recordings despite the varied use of AVID strategies (Fig. 4).

#### 3.2.3 Fasciculation frequency

**Fig. 5:**
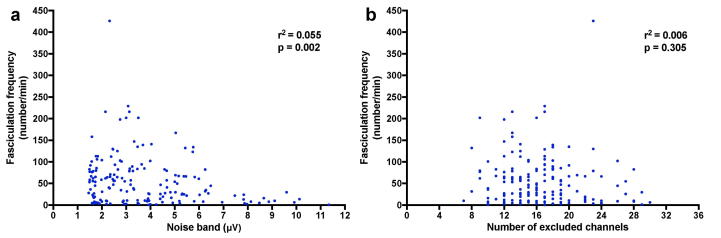
**Correlation between fasciculation frequency and data quality. a***Relationship between noise band and fasciculation frequency,* showing a very small, but significant (p < 0.05) coefficient of determination (r^2^). **b***Relationship between number of excluded channels and fasciculation frequency,* showing no significant correlation.

For all 165 recordings, the median fasciculation frequency was 40.5 per minute (10.6–79.4 IQR). Although there appeared to be a very small linear correlation between noise band and fasciculation frequency (r^2^ = 0.055, p = 0.002; Fig. 5a), we chose not to correct for this. Further data will be required to explore this potential correlation and to determine the need for standardisation between recordings of different noise levels. There was no linear correlation between the number of excluded channels and fasciculation frequency (p = 0.305; Fig. 5b).

## 4 Discussion

SPiQE was devised to take raw HDSEMG recordings of extended duration and produce consistently reliable outputs related to key fasciculation parameters, such as frequency, amplitude and inter-fasciculation intervals (De Carvalho and Swash, 2016a, Kleine et al., 2008, Drost et al., 2007, Bashford et al., 2019). We hereby introduce AVID, which provides the necessary framework to exclude one of the two principle sources of error: voluntary muscle activity (Li et al., 2018). In conjunction with the noise-responsive pipeline already described (Bashford et al., 2019), SPiQE provides unprecedented quantification capabilities in this clinical context.

The firing of fasciculations can be modeled as random, independent events following a Poisson distribution (Kleine et al., 2008, Fitzurka and Tam, 1999). It is known that the intervals between events in a Poisson distribution follow an exponential distribution (Ross, 2014). Therefore, where deviations between observed and expected inter-fasciculation intervals existed, the original assumption of random, independent fasciculation firing was violated. It’s well established that voluntary activity can be a cause of this, leading to excess intervals in the known range of motor unit recruitment (4–20 Hz; Fig. 3c-d) (De Luca et al., 1982, Broman et al., 1985). Multiplets are another cause (Sleutjes et al., 2015, Kleine et al., 2008), consistent with our observation of interval peaks < 50 ms (Fig. 3e-f), warranting future validation as a potential biomarker of disease progression. The third significant cause is the absolute refractory period of the neuronal action potential (Kleine et al., 2008), which can lead to a sharp fall in counts at very low intervals, as observed in this study amidst a high background fasciculation frequency (Fig. 3a). It is recognised that a firm mathematical basis enables the generation of more accurate and informative models, thereby enhancing interpretation of the observed data (Chen and Zhou, 2016).

For the 41% of recordings that were indicated to contain voluntary activity, we have introduced a practical guide to the selection of the optimal AVID strategy. The aim was to choose the strategy that was most specific at excluding epochs of voluntary potentials, thereby maximising the number of included fasciculation potentials. We took into consideration the processing time and performance for each strategy. In our experience, this minimal interaction by the user, making guided decisions from observed interval patterns, was a worthy compromise in an otherwise fully automated process. Although users would need to become familiar with these outputs, we anticipate that this would have a relatively shallow learning curve. Future validation of this approach’s inter-rater variability will be necessary.

The inclusion of a machine learning method was a key step in enhancing the precision of AVID (Zhang et al., 2014). Not only did it lead to lower processing times (an important consideration in the real world), it was the only AVID method reliably capable of identifying voluntary activity amidst higher background fasciculation frequencies. As the average frequency increased, the likelihood of inter-fasciculation intervals <250 ms also increased, thereby leading to falsely identified ‘voluntary’ blocks. In this scenario, an intelligent system was necessary to distinguish the true and false positives. It’s noteworthy that this model could be refined with more training data, aiming to improve its current accuracy of 86%.

It was important that the detection system prioritised sensitivity for voluntary potentials over specificity (Linden, 2006), as even the smallest epoch of voluntary activity could have a significant impact on the overall fasciculation count (Fig. 4b). Despite this priority, it was reassuring that only 1.5 minutes (out of 30) were excluded per recording, maximising the search for pathological features within the 28.5 minutes of analysable data. This exceeded the duration used for fasciculation analysis in previous studies using HDSEMG (Kleine et al., 2008, Sleutjes et al., 2016).

A limitation of these data was the large variation in noise levels between recordings (Fig. 5). It was unclear whether this was due principally to technical or biological factors, although the former seems most likely. Due to the relatively small data set in this study, further data will be needed to explore whether the small correlation between noise band and fasciculation frequency is real. There remains no doubt that recordings with higher noise levels should be interpreted with caution, highlighting the need to optimise the data acquisition procedure in order to improve skin-electrode contact and limit noise levels at source.

Fasciculations are captured by HDSEMG as spontaneous, short-lasting events with a high degree of morphological and temporal variability (Drost et al., 2007, Kleine et al., 2012). Large data sets are likely to be required to establish statistical trends amongst this complexity, a task simplified by the development of a robust and interactive tool like SPiQE. Its design has brought together basic signal-processing methods, established knowledge of motor neuron physiology and empirical relationships observed from serial recordings (De Carvalho, 2000, De Carvalho and Swash, 2016b). This foundation should lead to a deeper and more quantitative understanding of fasciculations, highlighting their relevance to motor neuron health in ALS.

## 5 Conclusion

We have developed a semi-automated method for the exclusion of voluntary muscle activity from HDSEMG recordings, which we have named AVID. It is an interactive tool that integrates seamlessly with the core data processing of SPiQE, allowing the user to set the desired AVID strategy at the beginning of each user command. We have demonstrated the practical advantages and analytical limitations of each AVID strategy, streamlining the decision-making process into a user-friendly guide for the purposes of maximising objectivity amongst users. SPiQE now has the sophistication to process raw thirty-minute recordings, leading to the automatic exclusion of error-prone periods. Minimal user interaction guides a series of well-defined steps based on simple readouts, making it a practical and suitable tool in the real world. We are in the process of making this tool available to other researchers as a standalone interface. When employed prospectively, we anticipate that SPiQE will lead to a more comprehensive awareness of the natural history of fasciculations in ALS and related disorders.

## Author contributions

JB set the study up and collected the data. JB and AW devised and tested the analytical technique. EM, MB, KM and CS supervised the project and provided expert technical and clinical guidance. RI provided expert input on statistical learning. JB wrote the article with editing from co-authors.

## Declaration of Competing Interest

None of the authors have potential conflicts of interest to be disclosed.
